# Field Performance of Nine Soil Water Content Sensors on a Sandy Loam Soil in New Brunswick, Maritime Region, Canada

**DOI:** 10.3390/s91109398

**Published:** 2009-11-24

**Authors:** Lien Chow, Zisheng Xing, Herb W. Rees, Fanrui Meng, John Monteith, Lionel Stevens

**Affiliations:** 1 Potato Research Centre, 850 Lincoln Road, P.O. Box 20280. Agriculture and Agri-Food Canada, Fredericton, New Brunswick, Canada; E-Mails: chowl@agr.gc.ca (L.C.); reesh@agr.gc.ca (H.W.R.); monteithj@agr.gc.ca (J.M.); lionels@agr.gc.ca (L.S.); 2 Faculty of Forestry & Environmental Management, University of New Brunswick, Fredericton, New Brunswick, Canada; E-Mail: fmeng@unb.ca (F.M.)

**Keywords:** sensor performance, soil water content sensor, statistical analysis

## Abstract

An *in situ* field test on nine commonly-used soil water sensors was carried out in a sandy loam soil located in the Potato Research Center, Fredericton, NB (Canada) using the gravimetric method as a reference. The results showed that among the tested sensors, regardless of installation depths and soil water regimes, CS615, Trase, and Troxler performed the best with the factory calibrations, with a relative root mean square error (RRMSE) of 15.78, 16.93, and 17.65%, and a r^2^ of 0.75, 0.77, and 0.65, respectively. TRIME, Moisture Point (MP917), and Gopher performed slightly worse with the factory calibrations, with a RRMSE of 45.76, 26.57, and 20.41%, and a r^2^ of 0.65, 0.72, and 0.78, respectively, while the Gypsum, WaterMark, and Netafim showed a frequent need for calibration in the application in this region.

## 1. Introduction

In the past few decades, a great number of automated techniques for point measurement of soil water content have been developed and tested because of the important role soil water content plays in guiding the management of irrigation and drainage [1-3]. Generally, those techniques can be classified into the following categories: (i) tensiometers, typically, a plastic tube with a porous ceramic cup attached at one end and a vacuum gauge or a pressure transducer on the other end, measuring the soil water tension or suction in units of kPa [4]; (ii) resistance blocks, two electrodes imbedded in a porous material such as gypsum, or a sand-ceramic mixture, determining soil water according to electrical resistance measured with an alternating current bridge [4,5]; (iii) heat dissipation type that derives soil water by measuring how much heat is dissipated in a ceramic medium buried in a soil [4,5]; (iv) dielectric sensors that obtain soil water by measuring the apparent dielectric constant of a soil [5]; (v) connector type sensors which are also based on dielectric properties of a soil, including: (a) Time Domain Reflectometry (TDR), measuring soil water content through measuring the travel time of an electromagnetic pulse along the metal rods of the waveguide, the time is determined by the soil bulk electrical permittivity which is strongly influenced by soil water, and (b) frequency domain reflectometers (FDR), similar to TDR but relying on reflected pulse reaching a set voltage rather than waveform analysis in TDR [5]; (vi) neutron method, consisting of a radioactive fast neutron source probe and a helium-3 detector, estimating soil water by the recorded count of thermal neutrons which are thermalized by the hydrogen present in the soil [3].

Although a certain amount of testing is normally conducted during the development of each sensor or some lab calibration is done by end users, due to the differences in design and functionality, each sensor may perform differently when used in real measurement operations in a specific region. The reliability of those tests is consequently limited by specific lab configurations and soil types [5-7] during test when applied beyond the test configurations.

Walker *et al.* [2] conducted in Australia an *in situ* comparison of several soil moisture sensors, including Environmental sensor Virrib (Environmental sensor Inc., San Diego, CA, USA; dielectric type), Campbell Scientific Inc. (CSI, USA, hereafter) CS615 reflectometer, and Soil Water Equipment Corporation Trase buriable- and connector-type TDR soil water sensors and found that among the tested sensors, the connector-type TDR performed the best against thermogravimetric measurements. The CS615 reflectometer yielded physically impossible soil water measurements during a period of soil water saturation. Leib *et al.* [8] did a similar field comparison of several soil moisture sensors including Irrometer Watermarks (The Irrometer Co., Riverside, CA, USA), EnviroScan (Sentek Environmental Technologies, Stepney, Australia), Troxler Sentry 200 (Troxler Electronics Laboratories Inc. NC, USA), AquaTel (Automata Inc., Grass Valley, CA, USA), AquaFlex (Streat Instruments, Christchurch, New Zealand), TRIME (MESA systems Co. Medfield, MA, USA), AquaPro (Aquapro-Sensors, Reno, NV, USA), and GroPoint (Environmental sensor Inc., San Diego, CA, USA; dielectric type), against soil water measurements obtained by a neutron probe calibrated for a Warden silt loam soil (coarse-silty, mixed, mesic, Xerollic Camborthids) under a crop of alfalfa. Their research concluded that connector-type TDR sensors produced soil water measurements within the ±2.5% (v/v) accuracy specified by the manufacturer when using the manufacturer's calibration relationship. Very recently, Zhao *et al.* [9] compared TDR, FDR, and a new type of soil water sensor based on standing wave ratio (SWR) in sandy loam and loam, loess, and peaty soils from Germany and concluded that SWR-based sensors performed better than both the TDR and FDR soil moisture sensors, regardless of soil type. In Hanson and Peters's research [10], the Trase (TDR instrument) and ThetaProbe (Delta-T, UK) were found to be reasonably accurate over a range of soil textures while the EnviroScan (Sentek Ltd., Australia) was inaccurate in silt loam and silty clay soils. These comparisons strongly suggest a performance variation of soil moisture sensors over different soil conditions tested.

Plauborg *et al.* [11] studied the impacts of both installation configurations (vertical and horizontal) and soil types on the performance of sensors such as the CSI Sensor CS616 (a later version of CS615), and Streat Instrument Aquaflex against TDR in different soil types. Their results demonstrated that the standard calibration from the factory needed to be corrected for sandy soils and that the CS616 sensor performed better with factory calibration in loam and clay soils. Although it is possible that using TDR as a reference may introduce some uncertainty because TDR may have measurement bias, their research demonstrated the variability of accuracy of each sensor when applied over different soils and with different installation configurations. Because of vertical variations of soil texture and water conditions, it is possible that the soil water sensors may perform differently in different soil layers from the top layer to the bottom layer down a soil profile although there is a lack of such information from the literature review.

Given the wide range of sensors and soil types covered in the above-mentioned inter-comparisons, it is safe to conclude that soil moisture sensors performed differently with soil types, different soil depths and different parts of a field [5,10]. Climate and soil physical conditions may be additional factors which directly or indirectly influence the sensitivity of sensors. For example, soil temperature is closely related to the conductivity and movement of soil water [12], which can significantly influence soil water measurements [3,5], in particular, measured by resistance sensors [13-15].

Obviously, a soil-specific calibration of each sensor under prevailing climatic conditions is a necessary prerequisite for a sensor to achieve its highest degree of absolute accuracy in soil water content measurements although not the only requirement [5,8,16,17]. However, the calibration conditions may not always be available. An inter-comparison of different sensors with factory calibration or minimum calibration would be very useful for the successful applications of sensors. On the other hand, as technology advances, improvements have been continuously brought to the soil water sensor market, making it relevant from time to time to investigate new or improved methods for *in situ* testing to obtain more knowledge than is available from the manufacturer's technical specifications [3]. Therefore, a carefully designed, *in situ* test and a field-based inter-comparison of sensors could provide valuable information for a wise application of soil moisture sensors. The Maritime Region of Canada has a very unique pattern of soil water distribution because of greatly variable weather conditions. An *in situ* evaluation of soil moisture sensors would assist in the application of soil water sensors for directing water management in this region.

The objectives of this study were to: (i) test the performance of several soil moisture sensors with factory calibration or minimum calibration in terms of different soil texture and soil water regimes; and (ii) provide experimentally-based suggestions on the application strategies of the sensors for obtaining acceptable measurements in the east coast Maritime Region of Canada.

## 2. Material and Methods

### 2.1. Methods to measure soil water contents

#### 2.1.1. Gravimetric method (GM)

Reference soil water content was determined by the gravimetric method and is expressed by volume as the ratio of the volume of water to the total volume of the soil sample. To determine the ratio for a particular soil sample, the water mass is determined by drying the sampled soil in an oven at a temperature of 100–110 °C (typically at 105 °C) to a constant weight and measuring the soil mass before and after drying. The water mass contained in the soil sample is the difference between the masses of the wet and oven-dry sample. The conversion of soil water content from a mass basis to a volume basis has applied knowledge of the bulk density of the soil and density of the water.

#### 2.1.2. Instrumental methods

Several types of soil water content instruments were installed in this exercise: (1) dielectric type, including Trase (6050X1; Soil Moisture Equipment Corp., Santa Barbara, CA, USA) and Moisture Point 917 (MP917; Environmental Sensors Inc., BC, Canada); Quasi-TDR based TRIME-FM (IMKO Micromodultechnik GmbH, Ettlingen, Germany); CS615 (Frequency domain reflectometer based, North Logan, UT, USA); (2) capacitance type Gopher (Soil Moisture Technology Pty Ltd., QLD, Australia), and Netafim (Netafim, Fresno, CA, USA); (3) Resistance type WaterMark (Irrometer Company, Inc., Riverside, CA, USA) and Gypsum blocks (Soil moisture technology Pty Ltd., QLD, Australia); and (4) Neutron type (Troxler 2651, Troxler Electronic Laboratories Inc., NC, USA). Without specific notes, the operation of each sensor strictly followed the instructions given by the instrument manual dispatched with the sensor.

**Figure 1.**
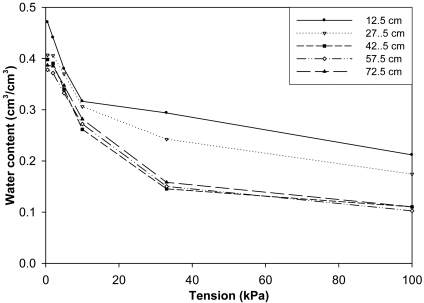
Partial soil water retention characteristic curves for different soil layers from the surface shown at the mid-point depth of the core. (a) 12.5 cm, (b) 27.5 cm, (c) 42.5 cm, (d) 57.5 cm, and (e) 72.5 cm.

For resistance type, the measured energy status of soil water was afterward converted into water contents by volume using the localized retention curve in Figure 1. The retention curve was determined as follows. Three relatively undisturbed soil cores (7.6 cm diameter × 7.6 cm high) were collected from depth intervals of 8.7–16.3, 23.7–31.3, 38.7–46.3, 53.7–61.3, and 68.7–76.3 cm with a double-cylinder, hammer-driven core sampler. The bulk densities were determined to be 1.19, 1.38, 1.41, 1.45, and 1.48 g/cm^3^, respectively. Prior to bulk density determination, the cores were used for partial water retention characteristic determinations. For tension ≤10 kPa (*i.e.*, 0.2, 2.0, 5.0, and 10 kPa) soil water contents were determined by a tension plate apparatus similar to that of Topp and Zebchuk [18]. A tension plate designed to handle individual cores was used to eliminate disturbance resulting from removing and weighing the core at the end of each tension setting. With this arrangement, water content of the soil at each tension setting was back calculated from the collected outflow. Water content at tension >10 kPa was determined by a pressure plate apparatus at 33 and 100 kPa settings using the same cores. Figure 1 was generated from these data. Bulk density was computed by dividing the oven-dry mass of the soil in the core by its volume.

### 2.2. Study site

**Figure 2.**
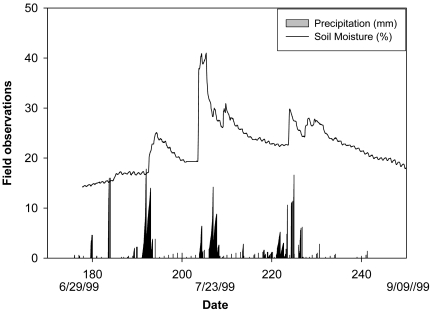
Precipitation and soil water content (obtained through CS615) at the depth of 30–35 cm during the sensor testing period (June to September 1999) on the research site, Potato Research Center, Agriculture and Agri-Food Canada, Fredericton, NB, Canada. The increased water content during day 203 was induced by irrigation using a sprinkler system.

The study site (45°55′13.2″ N, 66°36′33.7″ W) was located on the Agriculture and Agri-Food Canada Potato Research Centre in the Lower Saint John River Valley of New Brunswick. The soil was Orthic Humo-Ferric Podzol [19] developed on a coarse loamy ancient alluvium (fluvial) over coarse loamy morainal lodgement till [20], 30–100 cm of friable to very friable, yellowish brown (10 YR), acidic, sandy loam with 10–25% angular cobbles and gravels over a firm, dark brown (7.5 YR), acidic sandy loam with 10–20% angular gravels and some cobbles. It was characterized by 65 to 100 cm of friable topsoil over dense compact subsoil. The surface Ap horizon was a sandy loam with approximately 53% sand, 39% silt, and 8% clay by mass and relatively free of coarse fragments. Soil organic carbon content varied from 2.5% at the surface to less than 1% in the C horizon. Soil water content varied from 0.13 to 0.42 cm^3^ cm^−3^ and soil temperature from 15 °C to 27 °C at a depth of 30–35 cm during the testing period. The soil water condition and rainfall during the test period are shown in Figure 2.

### 2.3. Experimental design and sensor installation

Mass-based gravimetric soil water determinations (GM) were used as reference values for the other sensors in this research. Although the soil in the research site was a uniform sandy loam, to minimize the measurement error caused by variations in soil texture and bulky density between the GM site and other sensor sites, soil samples were collected from the soil nearby the instrumented location (within 5 m). Sensors for non-disturbed moisture determination were installed in an area of approximately 2 m in diameter. To reduce mass flow of soil water to the GM sampling holes, the holes were immediately refilled with soil from similar profiles taken from a site 10 m away. The GM sample sites were marked with flags so that no repeatedly soil-sampling at the same spot. Soil samples were taken with a MODEL L soil sampler which is a 91.44 cm one-piece welded unit with a replaceable screw-on tip on a 30.48 cm long sampling barrel, manufactured by Oakfield Apparatus Inc. (USA).

Corresponding to the depths of the installed sensors, duplicate sets of soil samples at depths of 5–20, 20–35, 35–50, 50–65, and 65–80 cm from the soil surface were taken on each measurement day. After obtaining the wet mass, the samples were oven dried at 105 °C for 24 hrs. Soil water contents by mass were calculated by dividing the difference between the wet and dry masses by the corresponding masses of dry samples. Water content by volume was then obtained by multiplying the water content by mass with the bulk density of the corresponding layer and then dividing it by the density of water.

In this research, CS615, WaterMark, and Gypsum probes were installed in a profile fashion with five layers at the above-mentioned depths. Two soil pits (approximately 1 meter in diameter and 100 cm in depth) were dug for ease of installing two sets of CS615 and Trase sensors. The length of the waveguide used in the CS615 and Trase were 30 and 20 cm, respectively and therefore, they were installed from the beginning of each depth interval at 30° and 50° angles, respectively to achieve the 15 cm resolution in depth. WaterMark and Gypsum were installed in duplicates at the mid-point of each depth interval by auguring a hole to the depth and placing the sensors. To ensure hydraulic continuity between the sensors and the surrounding soil, the sensors were pre-saturated by soaking in water and a small amount of slurry, a mixture of the soil surrounding the sensors and water, was then poured into the hole, around the sensor. MoisturePoint® (MP917) profiling probes (TypeH) capable of measuring soil moisture contents in 5 segments of 15 cm soil depths were inserted vertically from the soil surface to coincide with the depth intervals of other sensors.

For other profiling systems, thin wall access tubes made of either fiber-glass (Gopher and TRIME-FM) or aluminum (Troxler neutron) were inserted into the soil where the measurements were taken. The sensors were then manually lowered down to these access tubes and soil water contents were determined at the depths corresponding to the gravimetric and other sensor installation depths.

The CS615, Watermark, and Gypsum sensors were connected to a CR10X datalogger through a multiplexer for automated recording of either raw or processed data. The sensors recorded a 60-minute average of the measurements with a scanning interval of 10 minutes. There were no data gaps for those continuously operated sensors. After converting to soil water contents, an average of the measurements of the two replicates was calculated and used in further analysis. With exceptions of the CS615, Watermark, and Gypsum, other sensors were measured manually according to their operating instructions. These manual measurements were taken whenever gravimetric soil moisture samples were collected.

All sensors had two replications for each measurement depth except for TRIME which had four repetitions for each depth. Sensor orientation followed the standard rules described in the sensor manual. CS615, Trase, MP917, and TRIME-FM used a factory calibration, while other sensors were calibrated locally.

### 2.4. Statistical analysis

Following Leib *et al.* [8], three statistical parameters were adopted to assess the performance of each sensor against GM. The mean difference (MD), suggested by Addicott and Whitmore [21] to describe the average difference between sensor measurements and the corresponding GM measurements, is expressed as:

(1) $$\text{MD}=\frac{\sum_{i=1}^{n}\left(M_{si}-M_{gi}\right)}{n}$$

where *M_si_* is the *i*^th^ measurement obtained by a sensor, *M_gi_* is the *i*^th^ measurement obtained with GM, and n is the number of samples.

The relative root mean square error (RRMSE), proposed by Loague and Green [22] and calculated as the total difference between the sensor and the GM measurements of soil water content as a percentage of the mean GM measurement value, is given as:

(2) $$\text{RRMSE}={\left[\frac{1}{n}\sum_{i=1}^{n}{\left(M_{si}-M_{gi}\right)}^{2}\right]}^{0.5}\times (\frac{100}{M_{g}})$$

where *Mg* is the corresponding mean of gravimetric measurement, calculated as:

(3) $$M_{g}=\frac{1}{n}\sum_{i=1}^{n}M_{gi}$$

The coefficient of determination (*r*^2^), described in previous research [23,24], was used to display the degree of similarity between sensor measurements and GM measurements using the slope and intercept of the linear regression between the sensor measurements and the GM measurements. If the sensors performed well, the value of the MD and RRMSE should be close to zero, with a significant linear regression indicated by an intercept of zero, slope of 1, and *r*^2^ of 1. Other statistical analysis methods such as descriptive statistics and t-test were also used to examine the differences between sensor measurements and the GM measurements.

## 3. Results

### 3.1. Comparison between different sensor types

**Figure 3.**
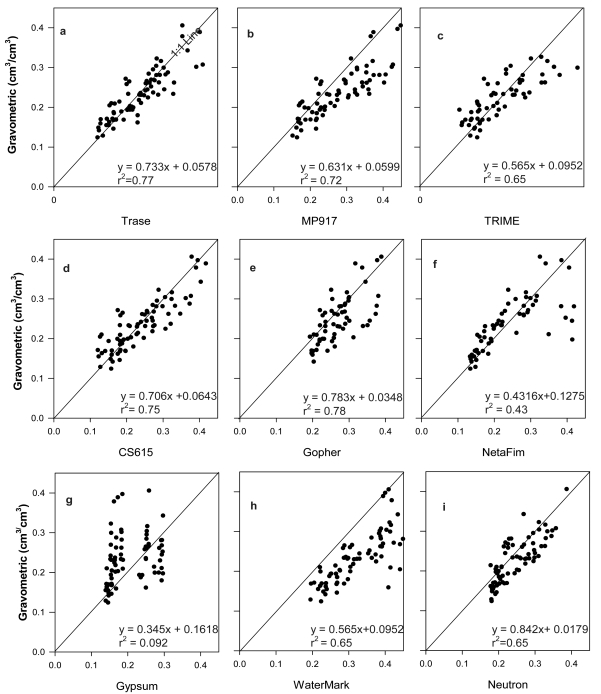
Regression relationship between gravimetric measurements (Y axis) of soil water and measurements obtained through different sensors (X axis). (The p- values of the regression between GM and each sensor measurement were <0.0001 except for Gypsum (0.008) and WaterMark (0.008).

Although the number of sensors in each sensor type was different, it was still possible to get a general comparison of sensor performance among the different sensor types. In this test we found that dielectric sensors (Figure 3a–d; r^2^ > 0.65, and slope ≥ 0.57, p < 0.0001) performed generally better than the resistance and capacitance sensors (Figure 3f–h; r^2^ < 0.65, and slope ≤ 0.57, p = 0.008), except for Gopher while the neutron sensor had average performance (Figure 3i, r^2^ = 0.65, slope = 0.84).

The scatter patterns of the sensor measurements against GM measurements displayed dependences on the sensor types. For example, the agreement between the measurements obtained with GM and the measurements with either the dielectric or neutron sensors (p < 0.0001) were normally higher than that between the GM measurements and the resistance sensor measurements (p = 0.008). The scatter dots followed 1:1 lines well, in particular, CS615 with a nice, tight distribution along 1:1 line (Figure 3d). Neutron also displayed a good distribution of scattered dots along 1:1 line but it was hard to evaluate when the moisture was great than 0.35 (cm^3^ cm^-3^) because of few data points available. It is obvious that WaterMark had most of the data points below the 1:1 line which suggests an overestimation (Figure 3i, p < 0.0001)). Gypsum showed unreasonable distribution pattern. Gopher had most of data points below 1:1 line (Figure 3e), an indication of overestimation. Further, the distribution range of Gopher measurements was significantly smaller than real data. There were some significant overestimations in Netafim (Figure 3f) too.

### 3.2. Sensor performance variations with measuring depths

**Figure 4.**
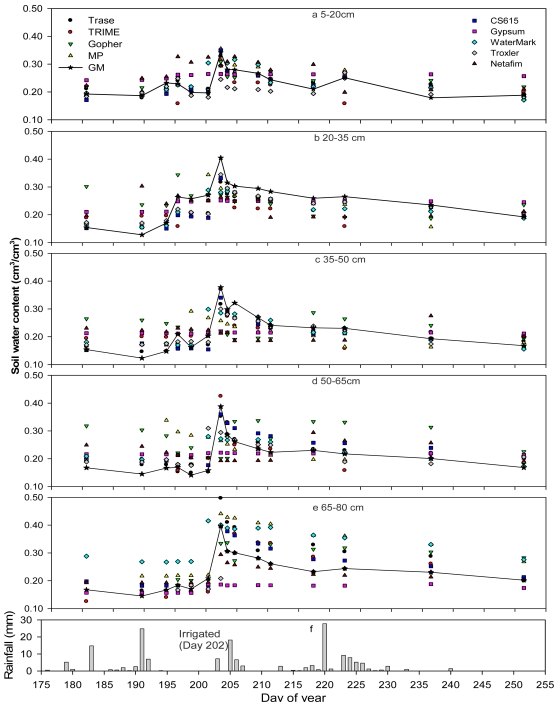
Time series of water contents obtained by different measurement methods as a function of soil depth.

**Figure 5.**
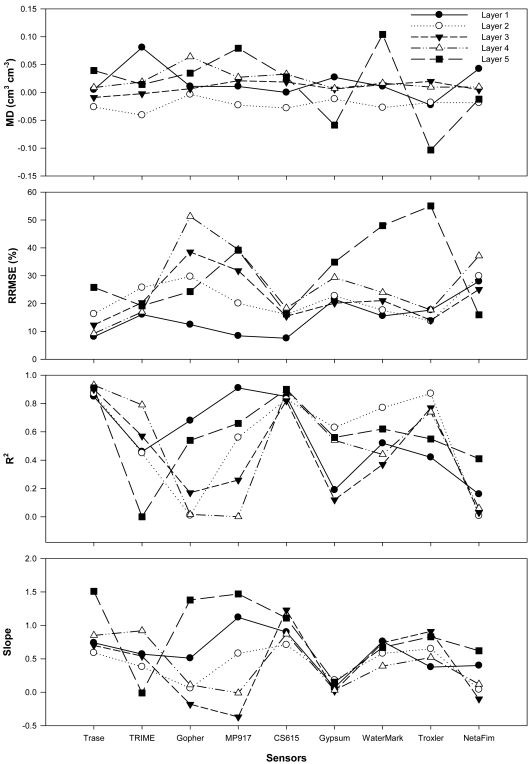
Comparisons of Mean Difference (MD), Relative Root Mean Square Error (RRMSE), Coefficient of Determination (*r*^2^), and regression slope obtained with different sensors at different soil layers with Gravimetric Method (GM) as reference.

All sensors generally followed the major trend of soil water content at different depths and reflected the impacts of rainfall pattern (Figure 4f) over time (Figure 4a–e). However, each sensor behaved differently at different soil depths (Figure 5), in particular, after irrigation.

In the soil layer 5–20 cm (Figure 4a), most sensors captured the major trend of soil water content except for Netafim and Gypsum. Gopher showed good performance, except for the last few data points. WaterMark and MP917 captured the main trend but overestimated soil water after an irrigation (July 21). Troxler showed some local offset (±5%) but generally followed the pattern well. Figure 5a,b suggest that TRIME, Netafim, and Gypsum generated larger MD values (positive value indicates overestimation and negative value underestimation) while the rest of the sensors displayed almost the same MD and RRMSE values. r^2^ values larger than 0.8 and close to unity slopes (Figure 5c,d) achieved in Trase, CS615, and MP917 suggests the best performance of these sensors in the top soil. The measurements from these sensors followed the pattern of water content very well at all times (Figure 4a).

In the soil layer between 20–35 cm (Figure 4b), all sensors performed generally well, except for a certain degree of offset occurring in NetaFim, Gypsum, Gopher, and TRIME (larger absolute values of MD and RRMESE values in Figure 5a,b, respectively, and lower r^2^ and slope farther away from unity in Figure 5c,d, respectively). From late June to middle July, Gopher and Netafim did not perform well and overestimated soil water contents. After an irrigation cycle on July 21, all of the tested sensors underestimated soil water content by 5–10%. However, Trase, CS615, and Troxler followed the trend better than the rest. A careful comparison before the heavy irrigation also suggested that CS615, Trase, and Troxler sensed soil water well, with only a slight overestimation. However, as Figure 5a indicates, underestimation occurred in this layer for almost all of the tested sensors based on the mean soil water content.

In the soil layer between 35–50 cm (Figure 4c), Gypsum, Netafim, and Gopher sensors performed very poorly. They did not capture the major pattern of soil water variation with time and rainfall pattern. WaterMark and MP only captured a part of the variation. However, Trase, CS615, and Troxler did well with only little offset (Figure 5a–d). In particular, CS615 worked very well.

In the soil layer between 50–65 cm (Figure 4d and Figure 5a–d), Netafim and Gopher performed unreasonably and overestimated soil water, MP917 failed to catch the trend, and Gypsum did not behave correctly. However, Trase, TRIME, Troxler, and CS615 generally performed reasonably well except for a small offset occurred in some dates during this test.

In the soil layer between 65–80 cm (Figure 4e), gypsum did not behave correctly and WaterMark consistently overestimated before the irrigation on July 21 while the rest of the sensors captured the soil water variation. However, after the irrigation, it seemed that most of the sensors did not have the capability to capture the soil water content in this layer and overestimated the soil water content (Figure 5a). CS615, Troxler, and Trase performed better although they also showed some offset after the heavy irrigation.

In general, the following can be concluded from this research: (i) Trase and CS615 performed the best among the tested sensors although they overestimated in deeper soil (depths >50 cm) and underestimated the soil water content in the shallow soil. However, the measurement offsets from these two sensors were relatively smaller. (ii) Troxler performed reasonably well in all soil layers, but slightly poorer than Trase and CS615. (iii) TRIME did not show consistently good performance in any of the soil layers. (iv) WaterMark, Gopher, MP917, and Netafim could not consistently capture the correct pattern of soil water contents, in particular, in the >50 cm layers.

Statistically, Trase was also found to perform the best among the tested sensors according to regression analysis (r^2^ = 0.77, slope = 0.733; Figure 3a) with a MD of −0.0058 and smallest RRMSE (Table 1) (Figure 4). A t-test displayed that the MD was not significant different from zero. However, the method underestimated soil water content by 2.7% even though median was very similar to that of the GM method.

Although the slope of the regression between CS615 measurements and GM measurements was less than 1 (0.706) with an intercept of 0.0643, and a r^2^ value of 0.75 (Figure 3d), the measurements obtained by the CS615 method displayed very small discrepancy with the GM measurements, with a relative bias of only −0.81% (Re; Table 2), and second smallest MD of −0.0058 (not significantly different from 0), and second smallest RRMSE of 16.93% (Table 1). Troxler performed slightly poorer than Trase and CS615.

WaterMark obtained the largest values in MD, RRMSE and Re (Tables 1 and 2). TRIME had a higher RRMSE than Trase, CS615, and Troxler but lower Re. The MD for TRIME is the smallest and is not significantly different from 0. MP performed averagely with regard to MD, RRMSE, and RE. Netafim overestimated soil water content by 8%, better than Gopher (17%) and Gypsum (11%).

## 4. Discussion

Several reasons could be responsible for the underperformance of the resistance type sensors in this test, including: (i) the resistance type had a calibration drift issue which is related to the configuration of the sensor [4], in particular, with continuous measuring (*i.e.*, in this research) and (ii) the resistance sensors were more temperature sensitive [3,4] and also were easily influenced by other factors beyond water content changes such as fertilizer scheme even with gypsum buffering [4,5].

Although the neutron method was found to perform better than the dielectric methods in previous research [8] and was suggested that a local soil type-based calibration of the neutron method could make this approach appealing [3], our research indicated that this method performed well but not as good as some of the dielectric methods such as CS615 and Trase. This finding is similar to the research finding by Hanson and Peters [11] who found that Trase was generally more accurate than a neutron method in all kinds of soils in their tests. Further, when using the neutron method as a reference to check the performance of other sensors, Leib *et al.* [8] found that the measured values of soil water varied significantly between sensor measurements and the calibrated neutron probe measurements. The conflicting results may be mainly caused by (i) soil types, (ii) local calibration for neutron, and (iii) soil water levels during the test period. Furthermore, the calibration requirement for neutron type sensor would involve additional work and sometimes is hard to carry out comparing with other water determination approaches in that calibration is not a requirement such as Trase, CS615 and TRIME-FM. The safety concern and required training with the neutron method also makes the method less favorable.

As indicated in Figure 5a, most sensors performed reasonably well between 20 and 50 cm depths, which is probably the major targeted soil layer of sensor design. The poor performance of the tested sensors on the surface and deeper layers may be attributed to large variations of soil water content (top layer) and water saturation condition (deeper layers).

Trase generally displayed the best performance with the factory-defined calibration coefficient in this research. With local calibration, the method could be expected to achieve a better accuracy. However, the sensor is the most expensive one among all tested sensors and also not very convenient in field operation when profile measurements and multi-point measurements are required [4,25] because the sensor is hard to be installed in a fashion of multiple locations.

Although CS615 sensor performed slightly poorer than Trase, the sensor is simple, light, and easy to handle in field work. Being easily connected to a datalogger, the sensor can be applied to obtain temporal and spatial measurements of soil water contents at the landscape level.

Troxler showed very promising performance in this region (Figure 3i) but poorer than the Trase and CS615. It overestimated soil water contents by 4%. Furthermore, it did not give consistently good performance at various depths (Figure 4 and Table 3). Similar to Trase, the sensor is large-sized, heavy and lacks flexibility to be used in the field. The high cost and strict safety requirements of application due to radiation makes this sensor less favorable in this region [4,14,25]. The calibration requirement makes this sensor inconvenient when the calibration conditions are unavailable. However, it is a good option to use this sensor as reference to either calibrate or check other sensors.

TRIME had a low relative bias of 1%, lowest MD, and a slight overestimation. However, the method displayed unstable performance, which may suggest necessity of local calibration.

MP demonstrated a below-average performance at varying depths. The method is hard to be automated.

Although the Gopher achieved the highest r^2^ and very high slope in regression analysis in this test, the scatter plot showed an unreasonably smaller range pattern which could significantly bias the method in terms of the time series, suggesting a calibration issue.

Netafim showed obvious estimation bias when water contents were higher than 0.3. Also the method performed poorly in regression analysis. The sensor performed better when the soil water content was lower than 0.3 but displayed a larger variation when the water content was above 0.4 although this problem seems to be common for the test sensors.

Despite Watermark and Gypsum following the major pattern of soil water content and being low cost to purchase they did not perform satisfactorily in this region. Increasing the number of duplications and careful and frequent calibration could possibly make these methods more acceptable [4,5] if continuous measurements must be made.

## 5. Conclusions

Among the tested sensors and regardless of installation depth and soil water regime, CS615, Trase, and Troxler performed the best with the factory calibration, with a relative root mean square error (RRMSE) of 15.78, 16.93, and 17.65%, and a r^2^ of 0.75, 0.77, and 0.65, respectively. These sensors can relatively consistently capture the variations of soil water content with the soil depths.

TRIME, Moisture Point (MP917), and Gopher performed slightly worse with the factory calibration with a RRMSE of 19.78, 26.57, and 20.41%, and a r^2^ value of 0.65, 0.72, and 0.78, respectively, while the Gypsum, WaterMark, and Netafim showed a need for careful calibration before application in this region, in particular, for making continuous measurements. Although Trase and Troxler demonstrated similar good performance as CS615 mostly, the higher cost and heavy package may constrain the application of these sensors.

## Figures and Tables

**Table 1.** MD and RRMSE of different soil water measuring methods.

	**Trase**	**MP**	**TRIME**	**CS615**	**Gopher**	**Netafim**	**Gypsum**	**Waterm**	**Troxler**
**MD**	−0.0058^a^	0.0436	0.0011^a^	−0.0058^a^	−0.0241	0.0152	−0.0279	0.0957	0.0110^a^
**RRMSE (%)**	15.78	26.57	19.78	16.93	20.41	32.55	32.51	47.34	17.65

a MD is not significantly different from zero at the 0.05 probability level using a *t*-test

**Table 2.** Statistics of volumetric soil water content (cm^3^/cm^3^) obtained by different methods.

**Items**	**Gravimetric**	**Trase**	**MP**	**TRIME**	**CS615**	**Gopher**	**Netafim**	**Gypsum**	**Waterm**	**Troxler**
Mean	0.232	0.226	0.2714	0.235	0.2302	0.271	0.2496	0.2055	0.3220	0.2412
Standard Error	0.008	0.008	0.010	0.010	0.010	0.007	0.013	0.008	0.010	0.006
Re (%)		−2.70	16.95	1.05	−0.81	16.80	7.56	−11.44	38.74	3.93
N	61	61	61	61	61	60	61	61	61	61

Mean is the average value of measurements obtained by different sensors during a period of 60 days (Julian day 195–251) in 1999. N is the number of days when the soil water contents were measured. Std. Error Means were mean standard error. Re were relative bias, calculated as Re (%) = (Sensor_mean_ − GM_mean_)/GM_mean_ × 100.
